# ASSESSING CANCER SCREENING DISPARITIES USING SECONDARY SURVEYS: CHALLENGES AND OPPORTUNITIES

**DOI:** 10.1093/geroni/igac059.588

**Published:** 2022-12-20

**Authors:** Preeti Zanwar

**Affiliations:** Jefferson College of Population Health, Philadelphia, Pennsylvania, United States

## Abstract

According to the WHO, cervical cancer is the fourth common cancer in women, with 90% each of the 604,000 new cases and 342,000 deaths in 2020 occurring in low-and middle-income countries. Cervical cancer can be cured if diagnosed early and treated promptly. Cervical cancer screenings by Pap-tests are evidence-based secondary prevention which are important in diagnosis and for receiving timely treatment for pre-cancerous lesions. I will present intersectional disparities in compliance with the U.S. Preventive Services Task Force guidelines for Pap testing in age-eligible women with disabilities by race/ethnicity using nationally representative Medical Expenditure Panel Survey. I find overall the proportion of women current with Pap testing is significantly lower among women with versus without disability. Additionally, I will provide example of other survey data such as the Behavioral Risk Factor Surveillance System that can be used for cancer screening and prevention and opportunities and challenges for using survey data.

